# Sustainable Recovery of Rare Earth Elements from Hard Disks: Grinding NdFeB Magnets and Financial and Environmental Analysis

**DOI:** 10.3390/ma18122697

**Published:** 2025-06-08

**Authors:** Paweł Friebe, Tomasz Suponik, Paweł M. Nuckowski, Marek Kremzer, Rafał Baron, Piotr Matusiak, Daniel Kowol

**Affiliations:** 1 KOMAG Institute of Mining Technology, 44-100 Gliwice, Poland; rbaron@komag.eu (R.B.); pmatusiak@komag.eu (P.M.); dkowol@komag.eu (D.K.); 2 Department of Geoengineering and Resource Exploitation, Silesian University of Technology, 44-100 Gliwice, Poland; tomasz.suponik@polsl.pl; 3 Materials Research Laboratory, Silesian University of Technology, 44-100 Gliwice, Poland; 4 Nanotechnology and Materials Technology Scientific and Didactic Laboratory, Silesian University of Technology, 44-100 Gliwice, Poland; marek.kremzer@polsl.pl

**Keywords:** NdFeB magnets, waste electronic equipment, planetary mill

## Abstract

Rare earth elements (REEs), particularly neodymium (Nd), dysprosium (Dy), and praseodymium (Pr), are critical in the production of neodymium–iron–boron (NdFeB) magnets used in electronic devices, wind turbines, and electric vehicles. Due to the limited availability of these metals, their recovery from waste electronic equipment such as hard disk drives (HDDs) offers a promising solution. The aim of this study was to develop a method to grind NdFeB magnets obtained from the physical recycling of HDD. The recycled magnets were ground using a planetary mill. A review of the literature highlights the limitations of the currently used grinding methods, which require energy-intensive pretreatment processes, specialised conditions, or expensive equipment. This study employed a Fritsch planetary mill, tungsten carbide grinding balls, and ethanol as a grinding medium. NdFeB magnet samples (120 g) were ground for different durations (0.5 h–15 h) at a speed of 300 rpm, using a cyclic operating mode to minimise material heating. The resulting powders were analysed using a laser particle analyser, an optical microscope, and an X-ray diffractometer. The results enable the determination of optimal grinding parameters, achieving an average particle size (d_50_) below 5 μm, which is essential for further processing and new magnet production. Finally, the economic and environmental aspects of producing the neodymium alloy were analysed.

## 1. Introduction

The data published in this article are part of work related to the recovery of rare earth metals from waste electronic equipment. This work draws on previous research experience related to HDD recycling in accordance with the circular economy [1,2,3].

Rare earth metals (REEs) play a crucial role in modern technologies, particularly in the production of neodymium–iron–boron magnets (NdFeB) [4,5]. These magnets mainly contain neodymium (Nd), dysprosium (Dy), and praseodymium (Pr) [6] and are widely used in electronic devices such as electric motors, generators, wind turbines, hard drives, speakers, drones, and electric and hybrid vehicles [6,7,8]. Due to their exceptional magnetic properties, NdFeB magnets have become indispensable in many sectors of the economy, leading to a growing demand for REEs [9,10,11].

In recent years, the European Commission has classified rare earth elements as critical raw materials due to their strategic importance and limited availability [12,13]. As a result, efforts must be made to reduce the dependence on external sources of these materials [14]. One of the emerging directions of obtaining REEs is their recovery from waste electronic equipment (WEE) containing NdFeB magnets [15,16]. An example would be the recovery of magnets from an HDD. 

The “Magnet-to-Magnet” method represents a promising approach in the recycling of NdFeB magnets [17]. It involves processing waste magnets to obtain a fine-grained neodymium alloy, which, after further metallurgical processing, can be used to produce new magnets [18,19]. A key step in this process is effective grinding of the alloy to the desired particle size of <5 µm [20,21].

**Figure 1:**
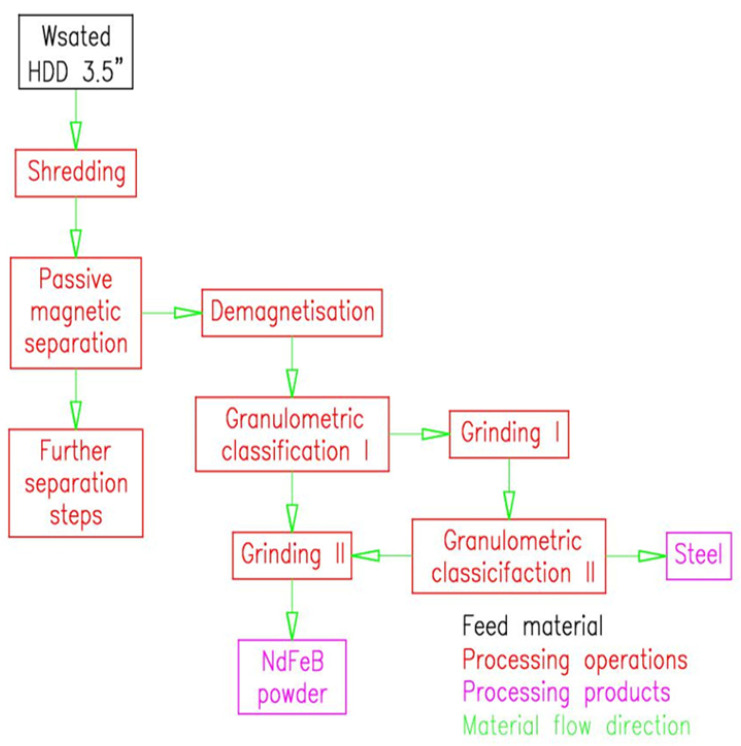
Method of recovering neodymium alloy and steel from 3.5” HDD.

HDD recycling technology, submitted to the Polish Patent Office [22], enables the recovery of various materials, including powdered neodymium alloy (<5 µm) and steel (Figure 1), as well as aluminium, stainless steel, plastics, and printed circuit boards, not analysed in this article and therefore invisible in the figure. The process begins with mechanical shredding of the HDD, during which larger components (e.g., metal casings, hard drive platters, PCB boards) are reduced to fragments of less than 35–40 mm in particle size, creating favourable conditions for subsequent processing operations. Magnetic separation was then performed using a steel plate to isolate the neodymium magnets along with the steel contaminants. The magnetic product is then directed to demagnetization, that is, heating the material above the Curie temperature characteristic of neodymium magnets, which is 350 °C [23], thus eliminating undesirable magnetic properties and allowing further comminution without interference. The subsequent step involves granulometric classification, aimed at separating larger steel particles. The remaining material, mainly composed of neodymium alloy, is selectively ground, ultimately producing particles less than 5 µm in size, which are crucial for the quality and final properties of neodymium magnets, mainly due to their influence on microstructure, magnetic anisotropy, coercivity, and the efficiency of the sintering process [21].

Various grinding methods for NdFeB magnets have been described in the literature, but each of them has limitations. McGuiness et al. [24] studied the effects of jet grinding (JM) on NdFeB material after initial hydrogen decrepitation (HD). This process allowed for the achievement of a very uniform particle size distribution with an average grain size of 2.3 μm, which is beneficial for the quality of magnetic powders. The authors emphasised that jet grinding enables achieving a uniformity difficult to obtain with conventional grinding methods. However, a drawback of this solution is the need for energy-intensive hydrogen decrepitation, requiring high pressure and temperature. Additionally, the lack of detailed data on grinding time and energy consumption complicates the evaluation of process efficiency.

Shdiara et al. [25] applied a hybrid ion grinding method to NdFeB magnets, involving bombarding the surface of the sample with argon ions. Smooth and uniform grains were obtained, as confirmed by scanning electron microscopy (FE-SEM) images. However, this method is costly because of the need for specialised equipment, vacuum, and technical gases. Additionally, the authors did not provide data on the obtained particle size distribution, which limits the practical applicability of this method on an industrial scale. Jurczyk et al. [26] investigated high-energy ball grinding of NdFeB alloys. This process led to the production of powders with grain sizes ranging from 12.5 μm to 0.3–1 μm, depending on the grinding time. The optimal particle size was achieved after 90 min, producing particles ranging from 0.5 to 4 μm. Although this method is time-efficient, it requires pre-grinding the material to a particle size below 0.2 mm and performing the process in an inert atmosphere to avoid oxidation. These additional steps increase the complexity and cost of the process, which could limit its use in recycling WEE with larger particle sizes. A review of the literature indicates a lack of grinding methods for NdFeB magnets that are simultaneously efficient, cost-effective, and do not require expensive preliminary processes. Existing techniques often require specialised conditions, such as high pressure, temperature, or protective atmospheres, which increase operational costs and hinder their application in industrial-scale recycling. Furthermore, many studies do not provide detailed information on process parameters, such as grinding time or energy consumption, making it difficult to compare and further optimise these methods.

Thus, a new method for grinding NdFeB magnets from WEE that is energy-efficient and cost-effective and does not require expensive preliminary processes is needed. The objective of this study is to present the results of research on the grinding process of NdFeB magnets using a planetary mill. This research focusses on optimising the grinding parameters to achieve an average particle size (d_50_) below 5 μm, which is crucial for the further processing and use of the material in the production of new magnets. The conducted research aligns with the principles of the circular economy, promoting the reuse of valuable resources and reducing dependence on REE imports [27]. The obtained results will contribute to expanding knowledge on effective and environmentally friendly methods for processing WEE containing NdFeB magnets, which is of significant importance for sustainable technological development.

## 2. Materials and Methods

### 2.1. Materials and Equipment

The test sample consisted of crushed neodymium–iron–boron (NdFeB) magnets obtained from HDD shredding according to the method shown in Figure 1. A total of 16 3.5-inch HDDs were first shredded in a disintegrator (shredder), designed at ITG KOMAG and fabricated by FUGOR Ltd. (FUGOR Sp. zoo, Krotoszyn, Poland) [3]. In passive magnetic separation, neodymium magnet particles were separated from other materials to be recovered in subsequent separation steps not presented in this article. This process is called passive because it takes place without the participation of external energy. A ferromagnetic steel plate measuring 10 × 10 cm with a thickness of 0.5 mm is employed in the process. The resulting material was then demagnetised, creating a demagnetised neodymium alloy. Demagnetisation was carried out using a muffle furnace(Nabertherm GmbH, Lilienthal, Germany) with a power rating of 2.0 kW. The process was conducted at a temperature of 350 °C for 40 min. Both the temperature and the duration were optimised, as lowering either parameter could result in incomplete demagnetisation of the material. Once magnetic induction was removed, the material proceeded to the first stage of granulometric classification, where the NdFeB alloy particles were separated from the steel impurities containing the residual NdFeB alloy in terms of particle size. The granulometric classification process was carried out using a Fritsch Vibratory Sieve Shaker ANALYSETTE 3 PRO (Fritsch GmbH, Idar-Oberstein, Germany) with a power rating of 0.2 kW, a sieve diameter of 200 mm, and a mesh size of 1 mm^2^. Next, the recovered NdFeB alloy containing steel was briefly ground, a step that selectively fractured the NdFeB alloy and facilitated its separation in the second stage of granulometric classification. The material was ground using a Testchem vibratory mill (TESTCHEM Sp. zoo, Pszów, Poland) with a power rating of 0.25 kW. The material was fed in portions of 120 g, with each grinding cycle lasting 2 s. A shorter grinding time resulted in insufficient comminution of neodymium alloy particles, increasing material losses, whereas a longer grinding time could lead to fragmentation of steel particles and contamination of the product. The process utilised the difference in grindability between the steel and the neodymium alloy. As a result, steel particles with a size of >1 mm were obtained, constituting the final product for sale, and neodymium alloy powder with a size of <1 mm, which was sent (together with products from granulometric classification I) for grinding in a planetary mill. A PULVERISETTE 6 Classic Line planetary mill manufactured by Fritsch GmbH (Fritsch GmbH, Idar-Oberstein, Germany) was used to carry out the grinding process. This device enables the intensive grinding of materials through the rotational motion of the grinding bowl around its own axis and around the base axis. The maximum rotational speed of the bowl is 650 rpm, but for the experiment, the speed was set to 300 rpm.

Ethyl alcohol with a chemical purity of 96% was used as the grinding medium. Ethanol acted as a friction-reducing agent between the particles and prevented surface oxidation of the material during grinding. A total of 60 g of ethanol was used for each sample, corresponding to a liquid-to-solid ratio of 0.5:1 (*w*/*w*).

Grinding balls with a diameter of 20 mm made of tungsten carbide (WC) were chosen as the grinding media due to their high hardness and wear resistance, which minimises contamination of the ground material with media particles. The grinding bowl, with a capacity of 250 mL and also made of tungsten carbide, ensured material compatibility with the media and reduced potential contamination.

In total, 120 g of material (research sample) obtained by this procedure was directed to a secondary grinding process, which was intended to produce NdFeB alloy powder with a particle size of less than 5 μm. Before the grinding process began, the samples were thoroughly mixed to ensure material homogeneity. No additional cleaning or magnetic separation processes were performed prior to grinding, in order to reflect the conditions of industrial recycling.

The NdFeB magnets contained in hard disk drives are characterised by high coercivity and remanence, and they consist primarily of neodymium (Nd), iron (Fe), and boron (B), with small amounts of other rare earth elements, such as dysprosium (Dy) and praseodymium (Pr). The chemical composition of the magnets influences their magnetic properties and the grinding process.

The particle size distribution was analysed using a laser particle analyser, the ANALYSETTE 22 MicroTec Plus (Fritsch GmbH, Germany), which provides precise analysis in the range of 0.08 μm to 2000 μm using a laser diffraction technique.

The structure of the material obtained after grinding was examined using a stereomicroscope, the SteREO Discovery, by Carl Zeiss AG (Jena, Germany). Phase analysis of the material was performed using an X-ray diffractometer, the X’Pert Pro MPD (PANalytical, Almelo, The Netherlands), equipped with a copper anode lamp and a PixCel 3D detector.

### 2.2. Methods for Grinding

The grinding process began by placing 120 g of fragmented NdFeB magnets in the grinding bowl along with 60 g of ethanol and 1200 g of tungsten carbide grinding balls, maintaining a mass ratio of the grinding media to the material of 10:1. Grinding was carried out in the planetary mill at a rotational speed of 300 rpm. Due to the excessive heating of the material that was observed, despite the use of ethanol, a cyclic mode of operation was introduced: after 5 min of grinding, a 10-min break was implemented, allowing the sample to cool. The experiments were conducted for net grinding times of 0.5, 1.0, 5.0, 10.0, and 15.0 h; these values refer solely to the cumulative periods during which the mill was actually running and therefore exclude the cooling pauses. After each net grinding-time interval, samples were taken for particle size distribution analysis.

At the end of the grinding process, the contents of the bowl were sieved through a mesh of the appropriate size to separate the grinding balls from the ground material. The ground material with ethanol was transferred to a container and dried in a laboratory dryer at 40 °C for 12 h to remove the remaining ethanol.

The particle size distribution was analysed using a laser particle analyser. A small amount (approximately 1 g) of the dried powder was suspended in deionised water with the addition of a dispersing agent, such as sodium hexametaphosphate, to prevent particle agglomeration. The suspension was subjected to ultrasound treatment for 5 min to ensure uniform particle dispersion. The sample was introduced into the particle analyser with a wet dispersion unit. For each sample, three measurements were made to ensure repeatability of the results. The analyser software calculated particle size distribution parameters, such as d_10_, d_50_, and d_90_.

The structure of the material obtained after grinding was examined using a stereomicroscope. A small amount of powder was placed on a glass slide with a drop of immersion oil to facilitate particle adhesion. The sample was covered with a cover slip, ensuring an even distribution of the particles. Observations were carried out at 1000× magnification, focussing on the shape, size, and uniformity of the particles. Microscopic images of a representative area of the sample were taken.

The phase composition of the material after grinding was determined by X-ray diffraction (XRD). The dried powder was pressed into a flat sample with a smooth surface to ensure uniform scattering of X-ray radiation. Measurements were performed using the X’Pert Pro MPD (Panalytical B.V., Almelo, The Netherlands) X-ray diffractometer, employing filtered Co Kα radiation with an Fe filter. Measurements were conducted in Bragg–Brentano (θ–2θ) geometry over the 2θ angle range from 10° to 100°, with a step size of 0.05° and counting time of 100 s per point. The obtained diffraction data were analysed using X’Pert HighScore Plus software, utilising the ICSD database of inorganic crystal structures. Phase identification was based on matching reference diffraction patterns to the measured data.

All experiments were performed in three independent repetitions to ensure the reliability and reproducibility of the results. The devices were calibrated according to the manufacturer’s instructions prior to the measurement series. Data from statistical analyses, such as mean values and standard deviations, were calculated using Microsoft Excel software.

### 2.3. Methods for Financial and Environmental Analysis

The literature analysis method was used to determine the prices of Nd, Pr, and Dy used in the construction of HDDs and their average content in them [28,29]. Information on the weight of disks collected by the three WEE collection companies located in the Silesian Voivodeship was used to assess the financial recovery of metals. 

The electricity demand was evaluated based on the process duration and the total rated power of the used devices. Additional environmental parameters that we tested were the level of sound pressure emitted by the devices and dust emissions into atmospheric air. Sound pressure was measured with a Trotec SL400 sound-level meter. Readings were taken every 30 s during device operation and then averaged. The measurement of dust emission was performed in accordance with the PN-Z-04008-07:1992 standard, using a 10 × 10 cm plate, with the results recalculated per square metre at a distance of 1 m from the dust emission source.

## 3. Results

### 3.1. Grinding of Neodymium Alloy

**Figure 2:**
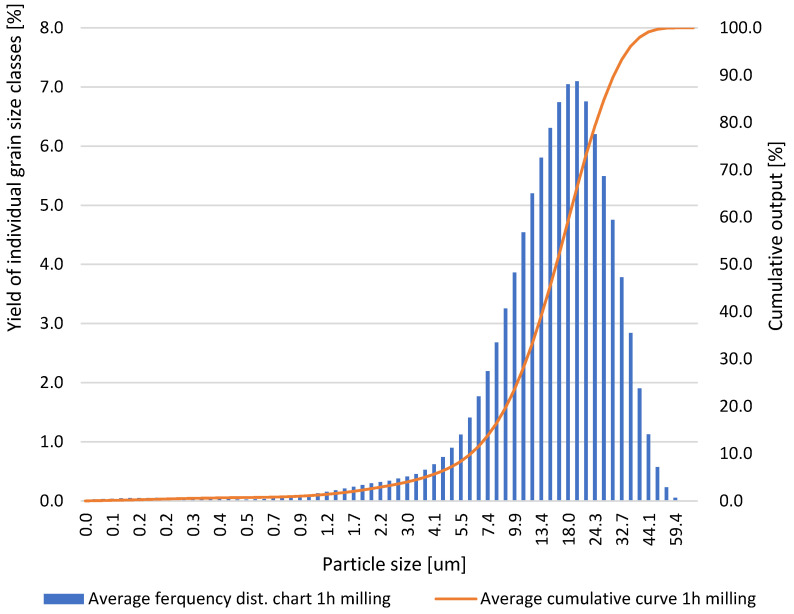
Average grain size distribution of the neodymium alloy ground for 1.0 h.

**Figure 3:**
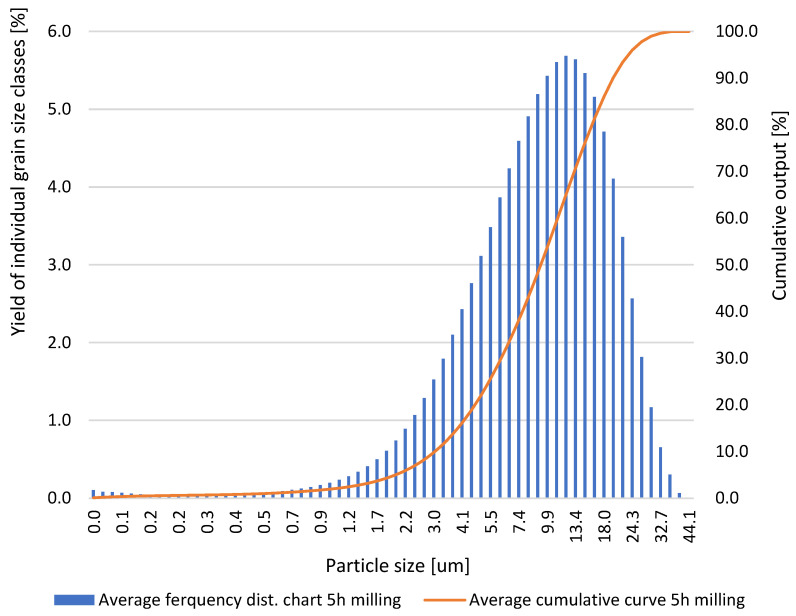
Average grain size distribution of the neodymium alloy ground for 5.0 h.

**Figure 4:**
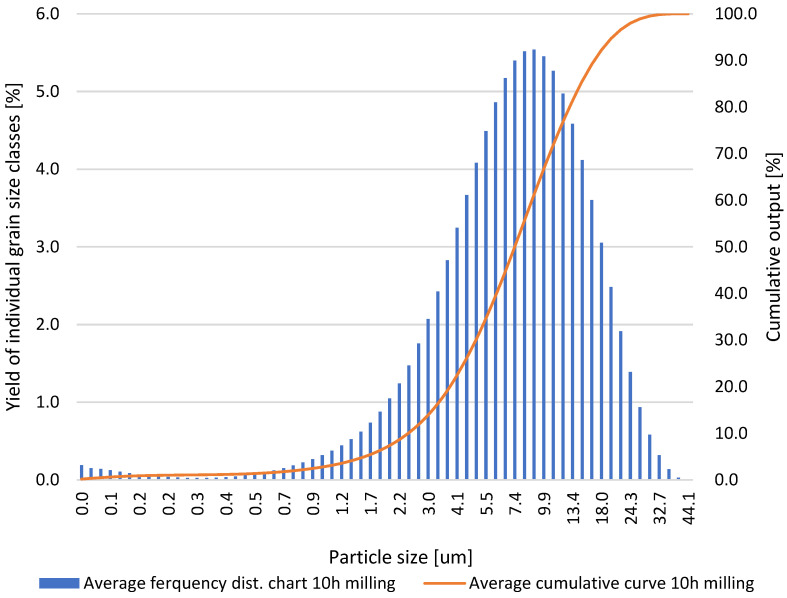
Average grain size distribution of the neodymium alloy ground for 10.0 h.

**Figure 5:**
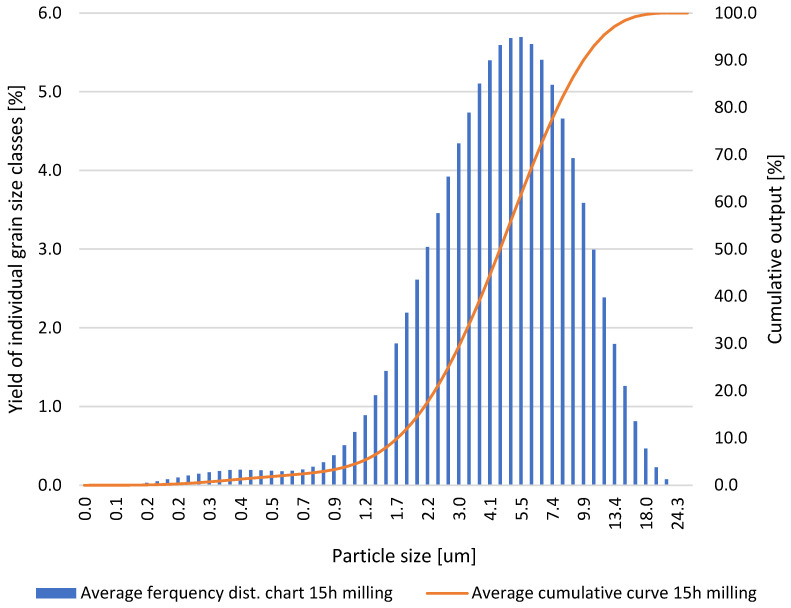
Average grain size distribution of the neodymium alloy ground for 15.0 h.

**Figure 6:**
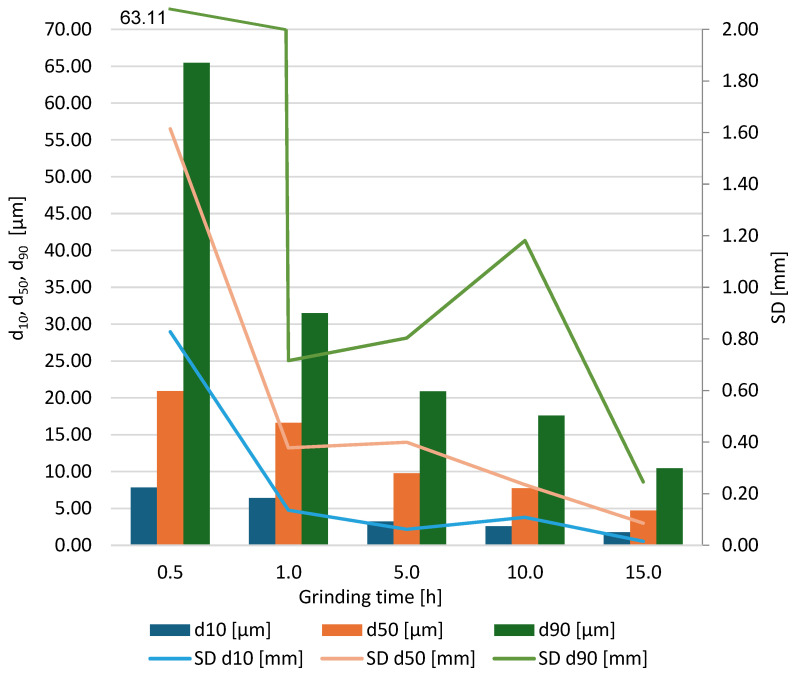
Column diagram of the parameters d_10_, d_50_ and d_90_.

**Figure A1:**
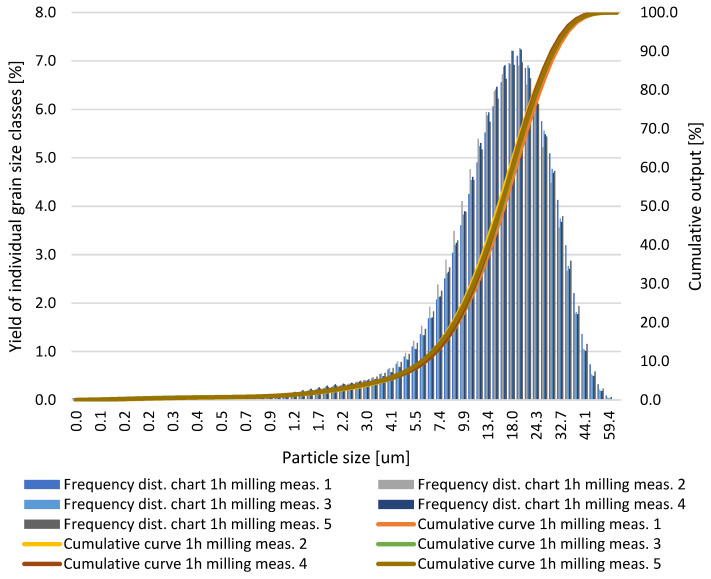
Sample after 1 h: individual frequency distribution charts and cumulative curves.

**Figure A2:**
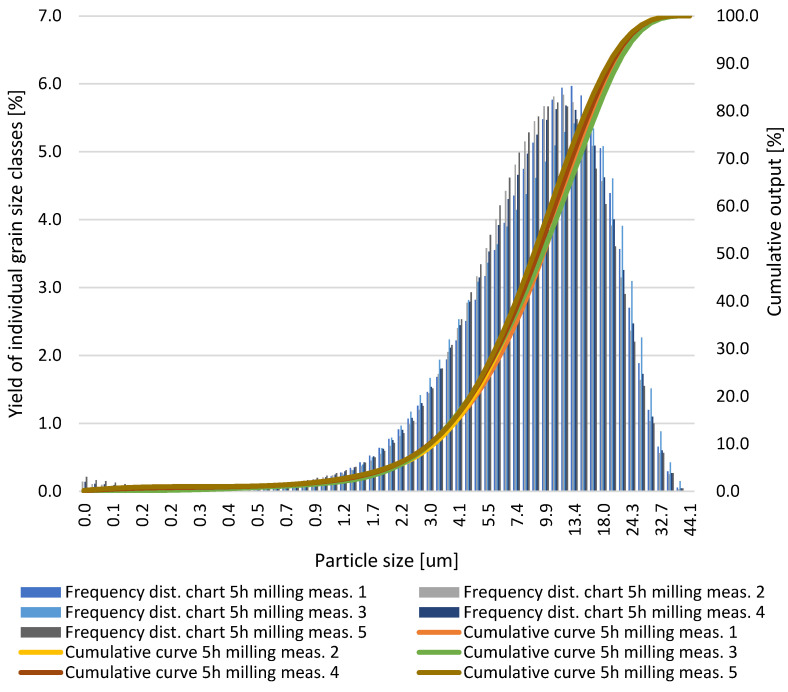
Sample after 5 h: individual frequency distribution charts and cumulative curves.

**Figure A3:**
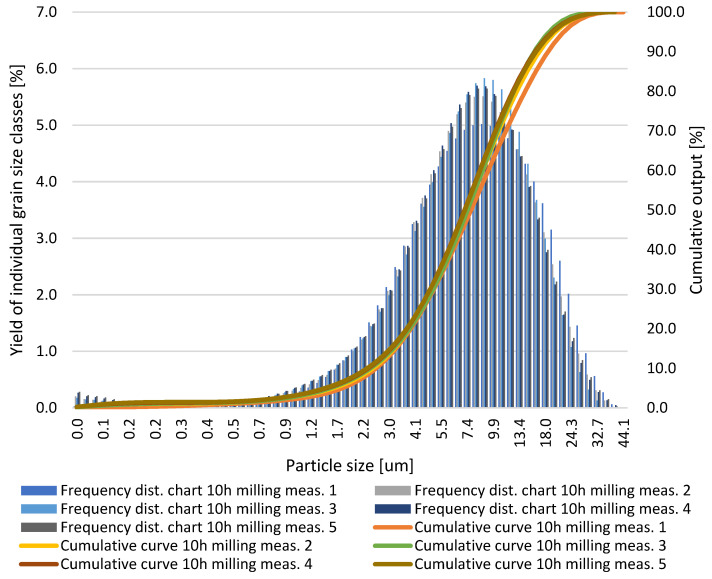
Sample after 10 h: individual frequency distribution charts and cumulative curves.

**Figure A4:**
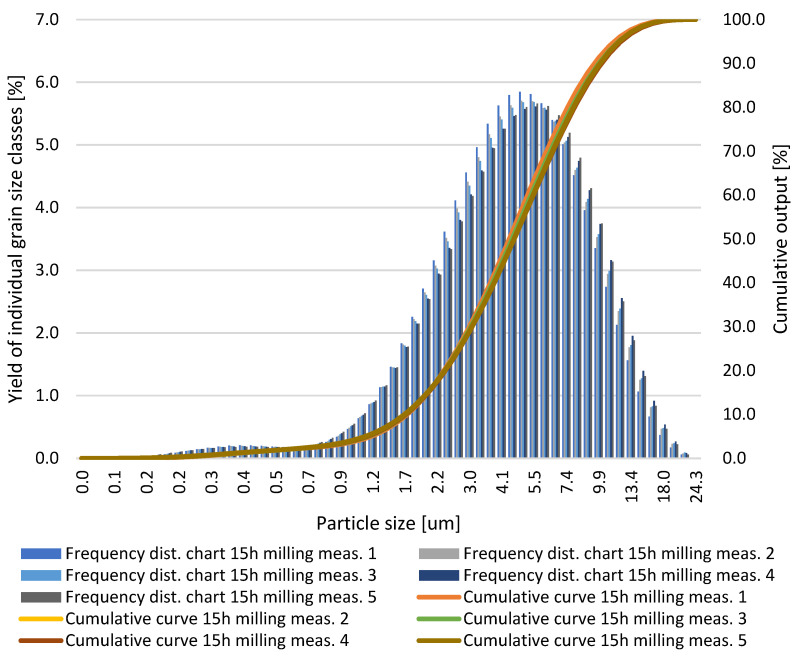
Sample after 15 h: individual frequency distribution charts and cumulative curves.

Based on the results obtained for the grinding times of the neodymium alloy of 0.5, 1.0, 5.0, 10.0, and 15.0 h, average column charts of frequency distributions and linear cumulative grain size distribution curves (Figure 2, Figure 3, Figure 4 and Figure 5) were drawn, along with a column chart that presents the obtained parameters d_10_, d_50_, and d_90_ for all used grinding times (Figure 6). Detailed data, including individual frequency distribution charts and cumulative curves for milling times from 1 to 15 h, are presented in Appendix A (Figure A1, Figure A2, Figure A3 and Figure A4). From the plotted curves, the parameters d_10_, d_50_, and d_90_ were read and are presented in Table 1, Table 2, Table 3, Table 4 and Table 5. The parameters refer to the grain size distribution of the grains and are used to describe the particle size in the sample. d_10_ denotes the particle size below which 10% of the aggregate mass lies. This parameter is an indicator for the finer fractions of the material. d_50_ refers to the particle size below which 50% of the aggregate mass lies, which means that half of the particles in the sample are smaller and half are larger. d_50_ represents the median particle size. d_90_ refers to the particle size below which 90% of the aggregate mass lies. This parameter is an indicator of the coarser fractions of the material. The parameters d_10_, d_50_, and d_90_ describe the particle size of the research material, which is crucial for analysing the products of the grinding process.

For a grinding time of 0.5 h, the individual outputs of the grain classes were not studied; only the parameters d_10_, d_50_, and d_90_ were determined.

**Table 1:** Parameters d_10_, d_50_, and d_90_ obtained via grinding the sample for 0.5 h.

Particle Diameters	Test 1	Test 2	Test 3	Test 4	Test 5
d_10_, μm	9.07	7.85	7.92	7.57	6.76
d_50_, μm	23.70	20.19	20.68	20.55	19.51
d_90_, μm	178.35	37.80	37.53	37.25	36.37

After 0.5 h of grinding, the following values were obtained: d_10_ ranged from 6.76 to 9.07 μm, d_50_ ranged from 19.51 to 23.70 μm, and d_90_ ranged from 36.37 to 178.35 μm.

**Table 2:** Parameters d_10_, d_50_, and d_90_ obtained via grinding the sample for 1.0 h.

Particle Diameters	Test 1	Test 2	Test 3	Test 4	Test 5
d_10_, μm	6.50	6.29	6.55	6.51	6.26
d_50_, μm	17.18	16.13	16.69	16.57	16.51
d_90_, μm	32.71	31.02	31.18	31.02	31.66

After 1.0 h of grinding, the following values were obtained: d_10_ ranged from 6.29 to 6.55 μm, d_50_ ranged from 16.13 to 17.18 μm, and d_90_ ranged from 31.02 to 32.71 μm.

**Table 3:** Parameters d_10_, d_50_, and d_90_ obtained via grinding the sample for 5.0 h.

Particle Diameters	Test 1	Test 2	Test 3	Test 4	Test 5
d_10_, μm	3.24	3.29	3.17	3.15	3.16
d_50_, μm	10.22	9.63	10.12	9.62	9.25
d_90_, μm	21.12	20.41	22.16	20.64	20.10

After 5.0 h of grinding, the following values were obtained: d_10_ ranged from 3.15 to 3.29 μm, d_50_ ranged from 9.25 to 10.22 μm, and d_90_ ranged from 20.10 to 22.16 μm.

**Table 4:** Parameters d_10_, d_50_, and d_90_ obtained via grinding the sample for 10.0 h.

Particle Diameters	Test 1	Test 2	Test 3	Test 4	Test 5
d_10_, μm	2.73	2.63	2.56	2.49	2.46
d_50_, μm	8.10	7.74	7.78	7.52	7.55
d_90_, μm	19.61	17.74	16.73	16.90	17.06

After 10.0 h of grinding, the following values were obtained: d_10_ ranged from 2.46 to 2.73 μm, d_50_ ranged from 7.52 to 8.10 μm, and d_90_ ranged from 16.73 to 19.61 μm. 

**Table 5:** Parameters d_10_, d_50_, and d_90_ obtained via grinding the sample for 15.0 h.

Particle Diameters	Test 1	Test 2	Test 3	Test 4	Test 5
d_10_, μm	1.78	1.77	1.76	1.76	1.74
d_50_, μm	4.58	4.67	4.69	4.79	4.78
d_90_, μm	10.06	10.39	10.46	10.72	10.57

After 15.0 h of grinding, the following values were obtained: d_10_ ranged from 1.76 to 1.78 μm, d_50_ ranged from 4.58 to 4.79 μm, and d_90_ ranged from 10.06 to 10.72 μm.

Detailed inspection of the differential and cumulative curves (Figure 2, Figure 3, Figure 4 and Figure 5) reveals that a distinct peak in the ultrafine region appears only after prolonged milling and shifts gradually as grinding proceeds. Specifically, the ultrafine mode is centred at ≈0–0.15 µm after 5 h, broadens to ≈0–0.20 µm after 10 h, and finally spans ≈0.15–0.55 µm after 15 h. Its absence after 1 h confirms that sub-micron particles were still negligible at the earliest stage. The emergence and progressive amplification of this peak demonstrate that extended milling steadily increases the share of the finest fraction. The appearance of these ultrafine peaks is a direct consequence of the characteristic comminution behaviour of the NdFeB alloy, whose grinding response favours the formation of very fine fragments once a critical level of mechanical activation is reached.

Table 6 indicates that as the grinding time increases, the values of the parameters d_10_, d_50_, and d_90_ decrease. This means that the grinding process effectively and gradually reduces the particle sizes. For example, the value of d_90_ decreases from 65.46 μm after 0.5 h of grinding to 10.44 μm after 15 h of grinding. This demonstrates a significant reduction in the average particle size as a result of prolonged grinding.

The uniformity coefficient U (Table 7) was calculated as the ratio d_90_/d_10_; values approaching unity indicate a narrow, uniform distribution, whereas larger values denote a broader span. During the first hour of milling, the rapid fragmentation of the coarse fraction lowers d_90_ and raises d_10_, causing U to fall sharply from 2.75 to 1.51. Between 1 h and 10 h, the continued generation of ultrafine particles (<2 µm) reduces d10 more quickly than d_90_, broadening the distribution and raising U to 1.94 even though S_50_ continues to rise, owing to the ongoing decline in d_50_ that is produced. After 15 h, the residual coarse particles have been largely eliminated; U decreases again to 1.85, whereas S_50_ reaches its maximum value, confirming that the milling process has achieved its highest degree of size reduction together with an effectively monomodal distribution. 

The comminution degree S_50_ (Table 7) increases monotonically and non-linearly with grinding time, rising from 34.9 after 0.5 h to 155.3 after 15 h. Because S_50_ is defined as the ratio of the median size of the feed (D_50_ = 0.73 mm) to the median size of the product (d_50_), each increment reflects the progressive decrease in the product median. The four-fold growth of S_50_ over the experimental window indicates that the median particle diameter was reduced by roughly an order of magnitude across the five intervals: from about 21 µm (0.5 h) to 17 µm (1 h), then to 9–10 µm (5 h), 7–8 µm (10 h), and finally below 5 µm (15 h). The largest relative gain occurs during the first five hours, when S_50_ more than doubles, confirming the high initial efficiency of planetary milling. Beyond ten hours, the rate of increase tapers off, showing that additional energy input yields progressively smaller reductions in median size as fracture approaches the material’s intrinsic limit. The final value of 155.3 demonstrates that the product median is nearly two orders of magnitude smaller than the feed median, corroborating the substantial refinement evident in the size–distribution curves and microscopy images.

**Figure 7:**
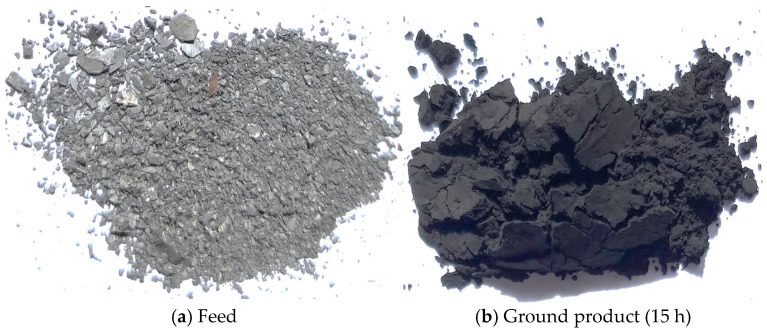
Presents macroscopic photographs of the material at two stages of processing.

Part (a) (Figure 7) shows the feed introduced into the planetary mill: irregular, angular fragments of metallic-grey NdFeB alloy ranging from sub-millimetre fineness to several millimetres in size, many of which retain a reflective lustre characteristic of freshly liberated magnet pieces. Part (a) (Figure 7) depicts the product obtained after 15 h of grinding, which forms a cohesive, matte-black powder agglomerate. The marked change in colour, loss of metallic sheen, and the absence of discernible coarse shards are consistent with extensive particle comminution.

**Figure 8:**
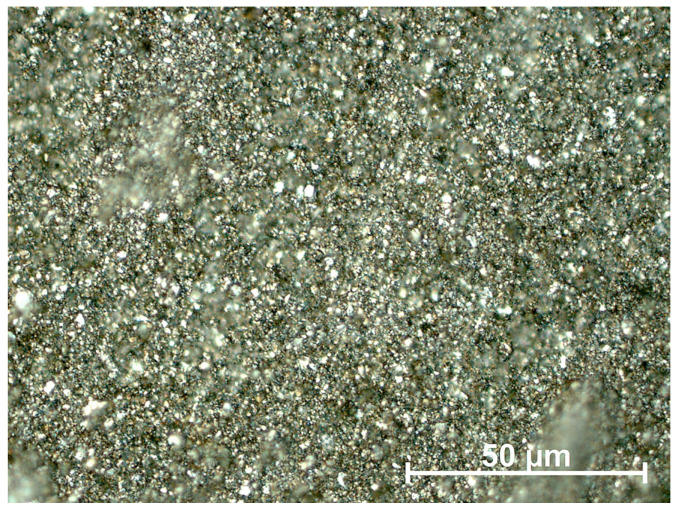
Ground product (15 h) at magnification.

Figure 8 displays a Zeiss Axio Observer Z1m microscope image of the powder obtained after 15 h of milling.

The field of view in Figure 8, delineated by the 50 µm scale bar, is densely populated by angular, faceted particles that are uniformly sub-10 µm in size, with no discernible oversized fragments. The speckled array of bright reflections originates from fresh fracture surfaces, indicating brittle comminution rather than ductile deformation. The absence of large voids or agglomerates confirms a high packing density and a homogenous dispersion of fines, consistent with the particle size statistics reported for this grinding interval.

**Figure 9:**
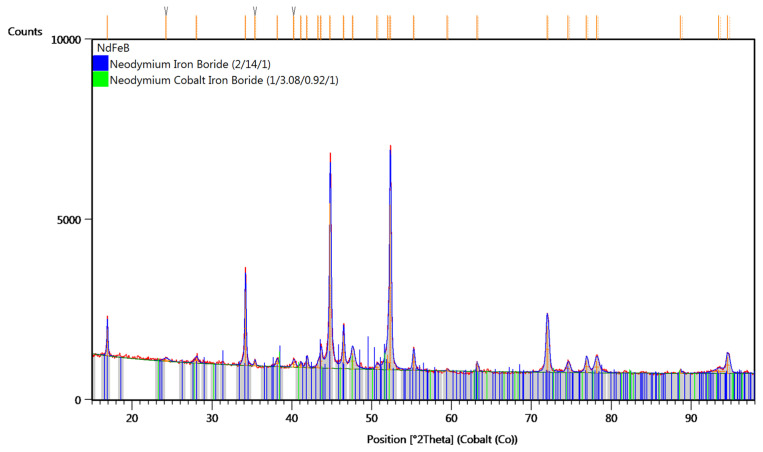
X-ray diffraction (XRD) pattern of the sample before the grinding process.

**Figure 10:**
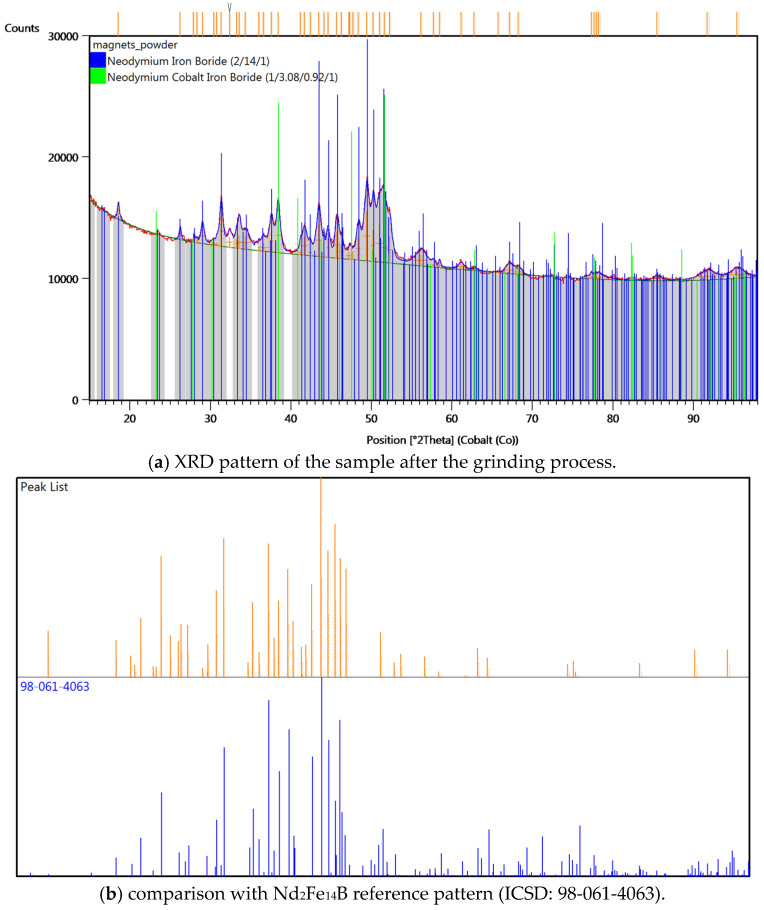
X-ray diffraction (XRD) analysis results.

The X-ray diffraction (XRD) pattern of the analysed material prior to grinding is presented in Figure 9. The diffractogram was recorded for the demagnetised NdFeB alloy before it was subjected to milling. For comparison, Figure 10 presents the XRD pattern obtained for the material after 15 h of grinding.

The results of the grinding tests of the neodymium alloy indicated that conditions were achieved that reduced the feed to a grain size d_50_ of less than 5 µm, while maintaining the homogeneity of the material. The observed decrease in the values of d_10_, d_50_, and d_90_ with increasing grinding time suggests a continuous fragmentation process, confirming the efficiency of planetary ball grinding in reducing particle size. Specifically, d_90_ decreased from 65.46 μm after 0.5 h of grinding to 10.44 μm after 15 h, highlighting the substantial reduction in particle size achieved. The uniformity index (U) analysis further supports the trend of increasing homogeneity over prolonged grinding durations. Initially, U was 2.75 after 0.5 h of grinding, dropping to 1.51 after 1.0 h. This initial decrease suggests a rapid homogenisation phase. However, subsequent grinding led to an increase in U to 1.82 (5.0 h) and 1.94 (10.0 h), followed by a slight reduction to 1.85 after 15.0 h. The final value suggests that an equilibrium state was reached in the particle size distribution, ensuring the desired level of uniformity of the material.

Microstructural observations provide additional confirmation of the effectiveness of the grinding process. Images obtained using a stereoscopic light microscope reveal a uniform particle distribution without the presence of larger distinguishable particles. The irregular morphology of the smaller grains suggests brittle fracture as the dominant fragmentation mechanism. The absence of agglomerates and macroscopic defects indicates that the applied grinding conditions yielded high-quality material, suitable for further processing.

The XRD analysis confirmed the presence of mainly Nd_2_Fe_14_B phase and NdCo_3_FeB in both the feed material and the product after grinding. XRD analysis results of magnets after milling show good line fit with the reference Nd_2_Fe_14_B phase pattern (ICSD: 98-061-4063). However, diffraction lines of the material before and after milling differ significantly in their intensity distribution. It can be assumed that this is the result of crystallographic texture occurring in the material created during the process of forming and sintering. This preferred orientation of crystallites was removed in the grinding process, and significant grain refinement was observed based on increased broadening (FWHM) of the diffraction lines. Although phase identification was successfully performed, it should be noted that XRD alone does not provide quantitative elemental composition or detect trace contamination. Therefore, complementary chemical analyses such as XRF, SEM (EDS) or ICP-OES are planned in future studies to evaluate the possible incorporation of elements such as W, Co, or C from the milling media and to verify the homogeneity and purity of the alloy.

These findings align with previous studies on the planetary grinding of NdFeB alloys [30,31], which have demonstrated similar trends in particle size reduction, microstructural refinement, and expected phase stability. The systematic decrease in d_50_ and the increase in homogeneity observed in this study are consistent with the expected results of prolonged high-energy grinding. The results suggest that the selected grinding parameters are optimal for producing fine, homogeneous NdFeB powder for potential reuse in magnet manufacturing or other applications.

Future research should focus on optimising grinding parameters to further improve efficiency while minimising energy consumption. Additionally, advanced characterisation techniques, such as scanning and transmission electron microscopy (SEM/TEM), should be employed to provide a more detailed analysis of particle morphology and potential nanoscale effects. Further investigations of the thermal and magnetic properties of the ground material would also be beneficial to assess its suitability for specific applications. Overall, the conducted study confirms the effectiveness of the grinding process in achieving the desired particle size and homogeneity of the material while preserving phase composition, thus providing a solid foundation for further material utilisation.

### 3.2. Economic and Environmental Aspects of Neodymium Alloy Production

Table 8 provides data on the amount of 3.5″ HDDs collected by three companies located in the Silesian province that collect WEE and the financial values of the metals recovered from them, mainly Nd, Dy, and Pr. An economic analysis of REE recovery from 3.5″ HDDs indicates the financial potential of this technology. The mass of HDDs collected per 1 million inhabitants in southern Poland was 13.6 t in 2024, and converted for Poland, 495.5 t. The average content of the neodymium alloy in HDDs is about 1%, giving a mass of 125.2 kg per 1 million inhabitants per year. Then, the mass of Nd, Pr, and Dy per 1 million inhabitants per year was 99.3, 15.0, and 10.9 kg, respectively. Taking the average values of Nd, Pr, and Dy [32,33] in 2025, the estimated value of recovered REEs from HDDs per 1 million inhabitants in southern Poland was USD 9607.04, while for a Poland of approximately 38 million inhabitants, it was USD 360,264.

Table 9 shows the environmental impacts of the unit processes of processing HDDs to produce neodymium alloy, based on the diagram shown in Figure 1.

An environmental impact assessment of technology unit operations revealed their different influences in terms of energy consumption, dust, and noise emissions. In the research work, it was assumed that the technology would not use process water in order to eliminate its treating and usage as well as drying of the resulting products. The highest energy consumption for 1 kg of feed per process was recorded in the last 15-h grinding stage. This amounts to 100.00 kWh/kg of neodymium alloy. However, there is little of this alloy in HDDs. When the energy consumption is normalised to 1 kilogram of recycled HDDs, then this value is 0.7692 kWh/kg of HDDs. The energy consumption for shredding, demagnetization, both granulometric classifications, and grinding I were 0.0880, 0.0857, 0.0041, and 0.000058 kWh/kg HDD, respectively. These operations showed significantly lower energy requirements. The total energy consumption for 1 kg of disks is 0.951158 kWh/kg HDD. The highest noise levels were recorded during planetary grinding and shredding, at 84.55 and 82.11 dBA, respectively. Dust emission to atmospheric air was at a similar level for shredding, granulometric classification, and grinding I, with the highest value recorded for grinding and amounting to 0.02 g/m^2^·h. 

These findings indicate that despite the energy intensity of the final grinding stage, the entire process can be considered relatively low-emission, provided that appropriate environmental protection systems are implemented. It is worth noting that there were no energy consumption or environmental emissions for passive magnetic separation. Thanks to this process, the neodymium alloy is separated from other materials in a cheap and ecological way. This process involves the use of a metal plate and a magnetic field induced by recycled neodymium magnets. For planetary grinding, no dust emissions were detected because loading and unloading of the laboratory mill vessel were performed inside a glove box.

Although this study concentrates on the grinding stage and the production of fine neodymium powder, the overall profitability of the patented recycling process is enhanced by the recovery of additional material fractions listed in the manuscript (e.g., aluminium, stainless steel, magnetic steel, plastics, and PCBs), all of which can be channelled to commercial reuse.

## 4. Conclusions

This study confirmed the high efficiency of the grinding process for NdFeB alloys recovered from waste electronic equipment, achieving a target particle size of d_50_ below 5 µm. The planetary ball grinding process led to a systematic decrease in particle size, with the most significant reduction occurring within the first five hours. After 15 h of grinding, the obtained d_50_ value of approximately 4.7 µm met the objective of this study.

Analysis of particle size distribution indicated a gradual improvement in material uniformity, as reflected in the homogeneity index (U), which stabilised around 1.85 after 15 h of grinding. Microstructural observations confirmed a fine-grained and homogeneous morphology, without visible agglomerates or macroscopic defects, which supports the effectiveness of the grinding conditions.

Analysis of phase composition using X-ray diffraction verified the presence of Nd_2_Fe_14_B and a smaller share of a cobalt-rich crystalline phase, consistent with the expected composition of the milled material. In summary, the optimised grinding process resulted in a uniform and defect-free material with the desired particle size distribution and confirmed presence of a mainly fine-grained Nd_2_Fe_14_B phase. These characteristics indicate that the obtained powder is suitable for further processing and potential applications in the development of new products.

The financial assessment showed the financial potential of REE recovery from HDDs, with the estimated mass of recovered Nd, Pr, and Dy per 1 million inhabitants of southern Poland per year being 99.3, 15.0, and 10.9 kg, respectively, which gives a total value of USD 9607 per year. The research results confirm that the recovery of neodymium alloy can be both economically profitable and environmentally friendly and sustainable. Total energy consumption for 1 kg of HDDs was 0.951158 kWh/kg HDD, and dust and noise emissions into the atmosphere were negligible. A comprehensive life cycle assessment (LCA) of the proposed recovery process, including cumulative energy demand and environmental impact across all unit operations, is planned in future studies to evaluate the sustainability of the method on a broader scale.

## Figures and Tables

**Table 6:** Summary table of average values and standard deviations for the parameters d_10_, d_50_, and d_90_ obtained via grinding the sample for 0.5, 1.0, 10.0, and 15.0 h.

	Grinding Time [h]									
Particle Diameters	0.5		1.0		5.0		10.0		15.0	
	Mean	SD	Mean	SD	Mean	SD	Mean	SD	Mean	SD
d_10_, μm	7.83	0.83	6.42	0.14	3.20	0.06	2.57	0.11	1.76	0.02
d_50_, μm	20.9	1.61	16.6	0.38	9.77	0.40	7.74	0.23	4.70	0.09
d_90_, μm	65.4	63.11	31.5	0.72	20.8	0.80	17.6	1.18	10.44	0.25

**Table 7:** Uniformity index and comminution degree obtained for sample grinding times of 0.5, 1.0, 5.0, 10.0, and 15.0 h.

	Grinding Time [h]				
	0.5	1.0	5.0	10.0	15.0
U, -	2.75	1.51	1.82	1.94	1.85
S_50_, -	34.9	43.9	74.7	94.3	155.3

**Table 8:** The amount of 3.5″ HDDs collected in 2024 and financial data on REE recovery from them (three WEE collection companies located in the Silesia region, while metal prices are based on [32,33]).

Mass of HDDs Collected per 1 Million Inhabitants of Southern Poland	Mass of Nd, Pr, and Dy in HDDs Collected per 1 Million Inhabitants in Southern Poland	Average Nd, Pr, and Dy Content in HDD per 1 Million Inhabitants in Southern Poland			Estimated Value of Nd, Pr, and Dy in HDDs per 1 Million Inhabitants of Southern Poland	Estimated Value Nd, Pr, and Dy in HDDs Collected in Poland in 2024
t/year	kg/year	kg/year			USD/year	USD/year
13.6	125.2	Nd	Pr	Dy	9607.04	360,264.00
		99.3	15.0	10.9		

**Table 9:** Environmental impact of HDD processing technology to produce neodymium alloy based on the diagram shown in Figure 1.

Process	Device Operation Time for 1 kg of Feed for Each Process in kg/h	Nominal Power in kW	Energy Consumption in kWh/kg Feed	Energy Consumption in kWh/kg HDD	Duste Mission, g/m^2^ h	Noise Emission, dBA
Shredding	62.85	5.54	0.0880	0.0880	0.015	82.1
Passive magnetic separation	93.36	-	-	-	-	-
Demagnetisation	0.18	2.0	5.8824	0.0857	-	-
Granulometric classification I	0.36	0.2	0.5348	0.0041	0.011	78.2
Grinding I	216.00	0.25	0.0075	0.000058	0.02	75.7
Granulometric classification II	0.36	0.2	0.5348	0.0041	0.01	77.7
Grinding II	0.008	1.0	100.00	0.7692	-	84.6

## Data Availability

The data presented in this study are not publicly available due to confidentiality and institutional restrictions.

## Appendix A

This appendix compiles the complete particle size distribution data for each grinding time, including all individual replicates. For clarity, each figure below presents five overlaid charts: a frequency distribution (left axis) and a cumulative undersize curve (right axis) corresponding to five independent measurements.

In the main text, only the averaged cumulative curve and the averaged frequency bars are shown for each grinding interval.
